# Computational Approach for the Development of pH-Selective PD-1/PD-L1 Signaling Pathway Inhibition in Fight with Cancer

**DOI:** 10.3390/cancers16132295

**Published:** 2024-06-22

**Authors:** Roderick C. McDowell, Jordhan D. Booth, Allyson McGowan, Wojciech Kolodziejczyk, Glake A. Hill, Santanu Banerjee, Manliang Feng, Karina Kapusta

**Affiliations:** 1 Department of Chemistry, Physics and Atmospheric Sciences, Jackson State University, Jackson, MS 39217, USA; roderick.mcdowell@yahoo.com (R.C.M.); kolodziejczyk.wojciech@gmail.com (W.K.); glakeh@icnanotox.org (G.A.H.); 2 Department of Chemistry and Physics, Tougaloo College, Tougaloo, MS 39174, USA; jdbooth@student.tougaloo.edu (J.D.B.); ajmcgowan@student.tougaloo.edu (A.M.); sbanerjee@tougaloo.edu (S.B.); mfeng@tougaloo.edu (M.F.)

**Keywords:** cancer, molecular docking, molecular mechanics, molecular dynamics, immunotherapy, PD1/PD-L1, natural inhibitors, pH-selective inhibition

## Abstract

**Simple Summary:**

Despite considerable progress in cancer research and treatment, cancer continues to be a major health challenge, often requiring invasive treatments with substantial side effects. Immuno-therapy, which targets the immune system’s PD-1/PD-L1 pathway, represents a promising alternative. This critical pathway allows cancer cells to avoid immune destruction by inhibiting T-cells. Our study employs computational techniques to develop inhibitors that block the PD-L1 pathway, specifically in the acidic environment of tumors. By analyzing around 10,000 natural compounds, we identified a potential pH-selective inhibitor that shows greater effectiveness in the acidic conditions typical of cancerous tissues. This research suggests a novel approach for experimental groups to explore, focusing on developing targeted, pH-dependent inhibitors that could mark a significant step in enhancing the precision and effectiveness of immunotherapy treatments, potentially revolutionizing cancer therapy.

**Abstract:**

Immunotherapy, particularly targeting the PD-1/PD-L1 pathway, holds promise in cancer treatment by regulating the immune response and preventing cancer cells from evading immune destruction. Nonetheless, this approach poses a risk of unwanted immune system activation against healthy cells. To minimize this risk, our study proposes a strategy based on selective targeting of the PD-L1 pathway within the acidic microenvironment of tumors. We employed in silico methods, such as virtual screening, molecular mechanics, and molecular dynamics simulations, analyzing approximately 10,000 natural compounds from the MolPort database to find potential hits with the desired properties. The simulations were conducted under two pH conditions (pH = 7.4 and 5.5) to mimic the environments of healthy and cancerous cells. The compound MolPort-001-742-690 emerged as a promising pH-selective inhibitor, showing a significant affinity for PD-L1 in acidic conditions and lower toxicity compared to known inhibitors like BMS-202 and LP23. A detailed 1000 ns molecular dynamics simulation confirmed the stability of the inhibitor-PD-L1 complex under acidic conditions. This research highlights the potential of using in silico techniques to discover novel pH-selective inhibitors, which, after experimental validation, may enhance the precision and reduce the toxicity of immunotherapies, offering a transformative approach to cancer treatment.

## 1. Introduction

Cancer still ranks among the top causes of death despite efforts devoted to developing treatments. This widespread illness, predominantly caused by acquired mutations in somatic cells, can affect any tissue and is defined by unchecked cell proliferation [1]. Treatment objectives might include anything from stopping the spread of cancer cells to curing the disease. Depending on the stage of the disease, frequent treatment options may include surgery [2], chemotherapy [3], hormonal therapies [4], radiation regimens [5], and immunotherapy [6]. The alternatives available today vary in terms of potential adverse effects and degree of invasiveness. The side effects of medication and cancer symptoms are frequently addressed [7]. Immunotherapy is one of the least invasive and promising options, which seeks to enhance the body’s defenses to destroy cancerous cells. The origins of cancer immunotherapy date back to early 1891, when William Coley proposed to utilize the immune system for cancer treatment while observing that combinations of live and inactive Streptococcus pyogenes and Serratia marcescens bacteria could lead to the shrinkage of tumors in patients with sarcoma [8]. Since then, immunotherapy for cancer treatment significantly improved, and substantial progress has been made in recent fundamental and clinical research [6,9,10]. Various approaches can be utilized for this type of treatment, including oncolytic virus [11,12]and cytokine therapies [13], cancer vaccines [14,15], adoptive cell transfer [16,17], and immune checkpoint inhibitor development [18].

**Figure 1:**
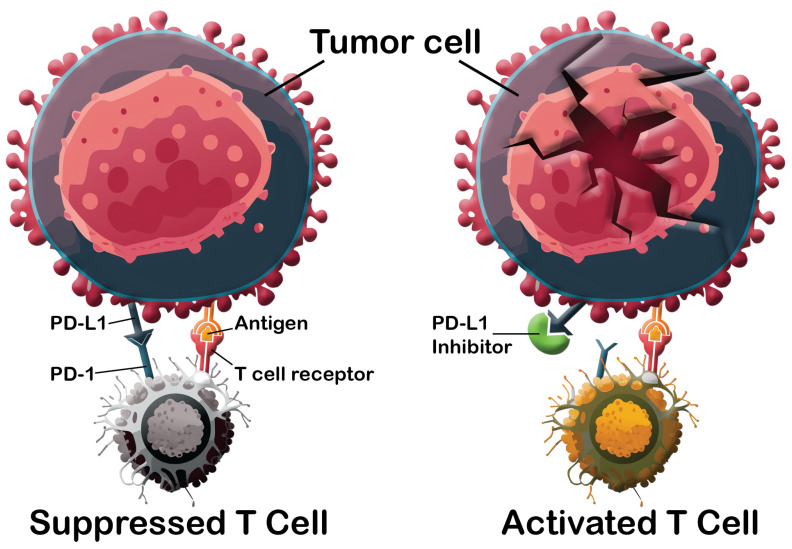
A schematic illustration of the T cell activation mechanism by inhibiting the PD1/PD-L1 immune checkpoint pathway.

The programmed cell death protein 1 (PD1) and programmed death-ligand 1 (PD-L1) are the main characters in the PD-1/PD-L1 immune checkpoint pathway. They are crucial to regulating immunological tolerance in the tumor microenvironment [19,20,21]. Generally, this pathway prevents overreaction and extensive inflammation in healthy cells by limiting T cell activation, proliferation, and cytotoxicity through the interaction of PD-1 on the activated T cells with PD-L1 or PD-L2 on the target cell. The cancer cells take advantage of this strategy by upregulating PD-L1/2 levels, resulting in so-called “cancer immune escape” [22]. Treatments may prevent this evasion by blocking PD1 or PD-L1, thus effectively reactivating T cells to target tumors (Figure 1). Along with the cytotoxic T-lymphocyte-associated protein 4 (CTLA-4) pathway [23], this pathway is a crucial target for novel cancer therapeutics, including the monoclonal antibodies (mAbs), peptides, and patented small molecules.

Currently, two FDA-approved mAbs for treating cancers target PD1: nivolumab [24] and pembrolizumab [25]. Several mAbs are in clinical trials that target different cancers and have shown promising anticancer results and safety profiles: AMP-224, AMP-514, and pidilizumab [26]. Disregarding their efficiency, the antibody treatments possess several drawbacks, such as high production costs, issues with stability, and the potential for triggering unwanted immune responses. Thus, the increased interest was focused on discovering small-molecule inhibitors of PD-1 and PD-L1. Most PD-L1 inhibitors act as dimerization agents and effectively dissociate a preformed PD-1/PD-L1 complex, as shown in [27] based on the example of Bristol Myers Squibb (BMS) biphenyl derivatives. The compound BMS-202 exhibited the highest efficacy among the series. Later on, several pharmaceutical companies discovered a series of small molecule PD-L1 inhibitors based on the same biphenyl core. Among the recent works, Biphenyl Ether and Oxadiazole Thioether-Based compounds were proposed as PD-1/PD-L1 inhibitors in [28], including a potent compound LP23, which showed 3.2-fold better inhibitory activity than the lead BMS-202 with an IC50 of 16.7 nM. Four protein-based designed small molecules showed promising results for lung and colorectal cancer models in both in vitro and in vivo assays [29]. A series of indanes were tested in [30], where compound D3 was found to be potent against PD-1/PD-L1 interaction (IC50 = 2.2 nM) and shown to induce immune activity of peripheral blood mononuclear cells (PBMCs) against MDA-MB-231 cells in a cell-based assay. Although many compounds based on the biphenyl scaffold have shown strong performance in assays measuring binding and disruption of the PD-1/PD-L1 interaction, not all demonstrate practical functionality in cellular environments. Additionally, some clinical evidence indicates the importance of accurately determining the dosage that yields optimal immune activation rather than increasing it to the maximum tolerated dose (MTD), which is the usual approach for many cytotoxic or targeted cancer medications [31]. Computational chemistry tools, such as molecular docking, molecular dynamics (MD) simulation, and the molecular mechanics approach, can offer a unique opportunity to investigate the binding mechanism [32,33] and to investigate new hits and potential inhibitors of the PD-1/PD-L1 [34,35,36,37,38].

In this work, we proposed an alternative approach for PD-1/PD-L1 pathway targeting. The tumor microenvironment becomes more acidic due to the buildup of protons and lactate. The acidic environment can promote immune evasion and tumor growth by increasing PD-L1 expression on tumor cells and suppressing immunological responses mediated by T cells expressing PD-1 [39,40]. Here, we propose the design of inhibitors that exhibit a higher affinity to PD-L1 in acidic conditions than in normal physiological pH conditions. This may reduce unwanted T-cell activation within the healthy cells while providing efficient immune response activation in tumor microenvironments. Different pH conditions may alter amino acids’ protonation states and protein secondary structures. This provides an opportunity for controlling the dynamics of binding and inhibition effectiveness; given the rise in pH-selective PD-1/PD-L1 pathway inhibitors, despite the challenges posed by tumor heterogeneity, where different regions within a tumor exhibit varying pH levels, targeting acidic environments remains effective [41]. Acidic regions are crucial for tumor survival and growth, making them strategic targets for therapy.

Here, various computational techniques were utilized in this investigation to design potential candidates with pH selectivity. This comprehensive approach aims to identify non-toxic, natural small molecules capable of obstructing the PD-1/PD-L1 binding site and sustaining T cell activation in the case of acidic tumor microenvironments, which is promising for cancer treatment.

## 2. Materials and Methods

To acquire the 3D structure of the PD-L1 protein, we retrieved the file with PDB ID 5J89 and PDB ID 8JBA from the RCSB Protein Data Bank (https://www.rcsb.org/ accessed on 1 January 2024). The protein structures were then carefully prepared using the Protein Preparation Wizard, implemented in the Schrödinger Software Package (Schrödinger Release 2024-1: Schrödinger, LLC, New York, NY, USA, 2024.). During the preparation phase, several optimization steps were implemented to ensure the reliability and accuracy of the protein structures. Water molecules were eliminated to focus solely on the protein’s relevant components. Chains C and D were removed from the tetrameric structure of a protein, and only chains A and B remained for further investigation in the case of 5J89. After these initial adjustments, the original hydrogen atoms were replaced, missing side chains were filled using Prime [42], and protonation states were generated at the target pH with Epik [43]. Further, the hydrogen bond network was minimized, and the structures were optimized using the OPLS4 force field [44]. The entire preparation process was repeated twice for two pH conditions: 5.5 and 7.4. By doing so, we ensured that the protein structures were accurately represented under different pH environments, which can significantly influence their behavior and interactions. A diverse library comprising 10,305 easily purchasable natural and genuine compounds from the extensive MolPort Database (https://www.molport.com/shop/access-databases accessed on 1 January 2020) was used for this investigation. To prepare these compounds for subsequent investigations, we employed the LigPrep tool by optimizing compounds and generating possible protonation states at two target pH values.

The screening of compounds was conducted using the Virtual Screening Workflow implemented in the Glide module [45]. This workflow enabled us to efficiently explore a vast library of compounds and identify potential candidates for further investigation. Grids were prepared centered on a co-crystallized ligand with a length of 36 Å and a size of the inner box of 10 Å. The scoring of potential inhibitors was carried out by employing the following algorithm: (1) initial High-Throughput Virtual Screening (HTVS); (2) Standard Precision (SP) for the top 10% hits from the HTVS; (3) Extra Precision (XP) for the top 10% hits from the SP. Ligands were docked flexibly with the OPLS4 force field. Virtual screening was repeated twice for protein and ligand structures prepared at pH = 5.5 and pH = 7.4. As a result of virtual screening, we identified and recorded the top 10 ligand candidates based on their scores for each pH condition. Following the screening process, we identified compounds that demonstrated successful scores at a pH of 5.5 but did not perform as efficiently at a pH of 7.4. Five selected candidates were subjected to further simulations using the MM/GBSA (Molecular Mechanics/Generalized Born Surface Area) module. The MM/GBSA [46] is a widely employed method for estimating binding free energies in molecular systems, enabling more accurate scoring than molecular docking. It combines molecular mechanics calculations, which account for the energy contributions of bonded and non-bonded interactions within the molecule, with the Generalized Born (GB) solvent model that captures the solvation effects. The calculations were carried out for the five selected compounds complexed with the protein structures at both pH values utilizing the VSGB solvation model and OPLS4 force field. Protein flexibility was enabled for residues at a distance of up to 12 Å from all ligands processed. Through these calculations, we obtained refined estimates of the compounds’ binding free energies, enabling further, more accurate scoring.

After identifying the top five molecules with the most favorable binding affinity at low pH, we performed ADMET (Absorption, Distribution, Metabolism, Elimination, and Toxicity) profiling on these compounds to compare their predicted pharmaceutical properties with the lead BMS-202 and novel LP23 inhibitors. The isomeric SMILES codes for each compound were retrieved from the MolPort database (https://www.molport.com/shop/index accessed on 1 May 2024) or the PubChem database (https://pubchem.ncbi.nlm.nih.gov/ accessed on 1 May 2024):

*Compound* **1** *(MolPort-001-741-806):*

O[C@@H]1[C@@H](COC(=O)c2cc(O)c(O)c(O)c2)O[C@@H](OC(=O)c2cc(O)c(O)c(O)c2)[C@H](O)[C@H]1OC(=O)c1cc(O)c(O)c(O)c1

*Compound* **2** *(MolPort-005-945-958):*

CC(C)(O[C@@H]1O[C@H](CO)[C@@H](O)[C@H](O)[C@H]1O)C(OC(=O)C=Cc1ccc(O)cc1)C(=O)OCc1ccc(O[C@@H]2O[C@H](CO)[C@@H](O)[C@H](O)[C@H]2O)cc1

*Compound* **3** *(MolPort-001-741-210):*

Oc1cc(cc(O)c1O)C(=O)OC[C@H]1O[C@@H](OC(=O)c2cc(O)c(O)c(O)c2)[C@H](OC(=O)c2cc(O)c(O)c(O)c2)[C@@H](OC(=O)c2cc(O)c(O)c(O)c2)[C@@H]1OC(=O)c1cc(O)c(O)c(O)c1

*Compound* **4** *(MolPort-001-740-310):*

[#6]-[#6@@H]-1-[#8]-[#6@@H](-[#8]-[#6]-[#6@H]-2-[#8]-[#6@@H](-[#8]-[#6][#6]=[#6](/[#6])-[#6]-[#6][#6]=[#6](/[#6])-[#6]-[#6][#6]=[#6](/[#6])-[#6]-[#6][#6]=[#6]([#6])-[#6])-[#6@H](-[#8]-[#6@@H]-3-[#8]-[#6@@H](-[#6])-[#6@H](-[#8]-[#6](-[#6])=O)-[#6@@H](-[#8]-[#6](-[#6])=O)-[#6@H]-3-[#8]-[#6](-[#6])=O)-[#6@@H](-[#8])-[#6@@H]-2-[#8])-[#6@H](-[#8])-[#6@H](-[#8])-[#6@H]-1-[#8]

*Compound* **5** *(MolPort-001-742-690):*

CC(CO[C@@H]1O[C@H](COC(=O)C=Cc2ccc(O)c(O)c2)[C@@H](O)[C@H](O)[C@H]1O)C1(O)COC(=O)C1

*BMS-202:*

CC1=C(C=CC=C1C2=CC=CC=C2)COC3=NC(=C(C=C3)CNCCNC(=O)C)OC

*LP23:*

CC1=C(C=CC=C1OCC2=NN=C(O2)SCC3=CC=CC(=C3)CNC(CO)C(=O)O)C4=CC=CC=C4

SMILES codes were used as inputs for ADMET prediction utilizing the ADMETLab 2.0 website (https://admetmesh.scbdd.com/service/evaluation/index accessed on 1 May 2024). ADMET Evaluation tab was used to identify molecules with favorable pharmacokinetic profiles and lower toxicity risks, paving the way for more efficient and safer drug development. This website provides an easy approach to comprehensively and efficiently predict ADMET profiles for chemicals. Predictive models were built on a high-quality database of a quarter million entries across 53 endpoints with a multi-task graph attention framework [47]. The following properties were assessed: Lipinski Rule, Pfizer Rule, human colon adenocarcinoma cell line (Caco-2) permeability, Madin−Darby canine kidney cell (MDCK) permeability, P-glycoprotein substrate, human intestinal absorption (HIA), blood–brain barrier (BBB) penetration, fraction unbound in plasma (Fu), metabolism inhibitors and substrates, human ether-a-go-go-related gene (hERG) blockers, human hepatotoxicity, drug-induced liver injury (DILI), AMES toxicity, skin sensitization, carcinogenicity, and respiratory Toxicity.

**Figure 2:**
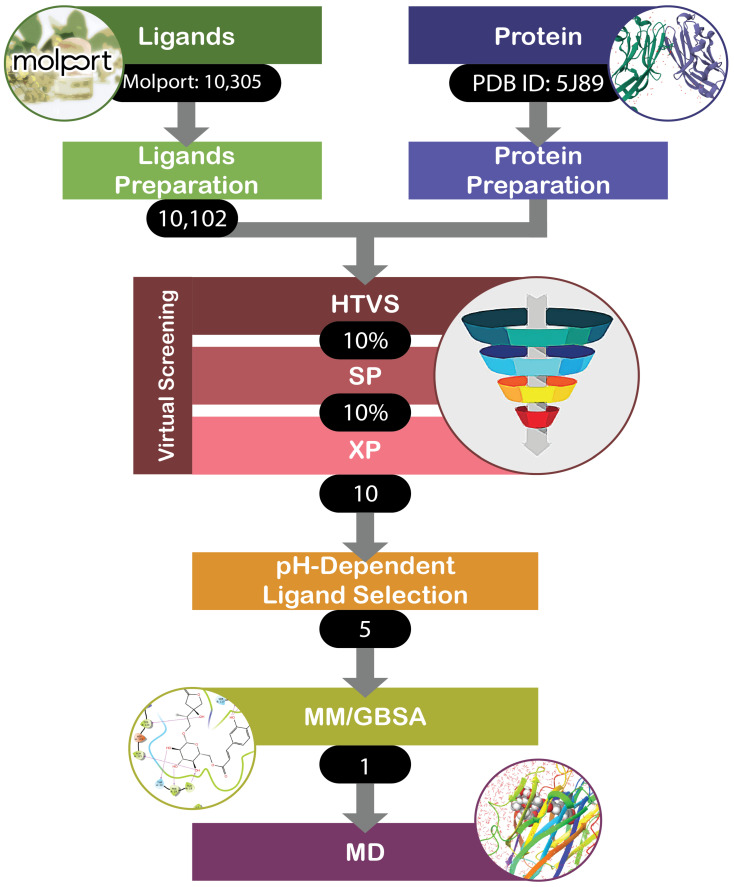
Algorithm of approaches for pH-dependent drug discovery in this work.

The most successful candidate was selected as the one that exhibited significantly higher binding affinity toward the PD-L1 dimer at pH = 5.5 compared to pH = 7.4. The chosen ligand–protein complexes, refined with MM/GBSA at both target pH conditions, were further subjected to molecular dynamics simulation with the Desmond module [48]. The System Builder was used to build an orthorhombic box of minimal size solvated with single-point charge (SPC) water molecules around the complexes. Systems were neutralized with four sodium cations for the case of pH = 7.4 and five chlorine anions for pH = 5.5. The OPLS4 force field was used for all the simulations. Systems were subjected to the standard eight-step relaxation protocol followed by the 1000 ns actual run with a 25 ps recording time step using the NPT ensemble class. We used the Simulation Interaction Diagram to analyze the obtained MD trajectories. The generalized algorithm for the proposed approach is sketched in Figure 2.

## 3. Results

### 3.1. Benchmark and Positive Control

**Figure 3:**
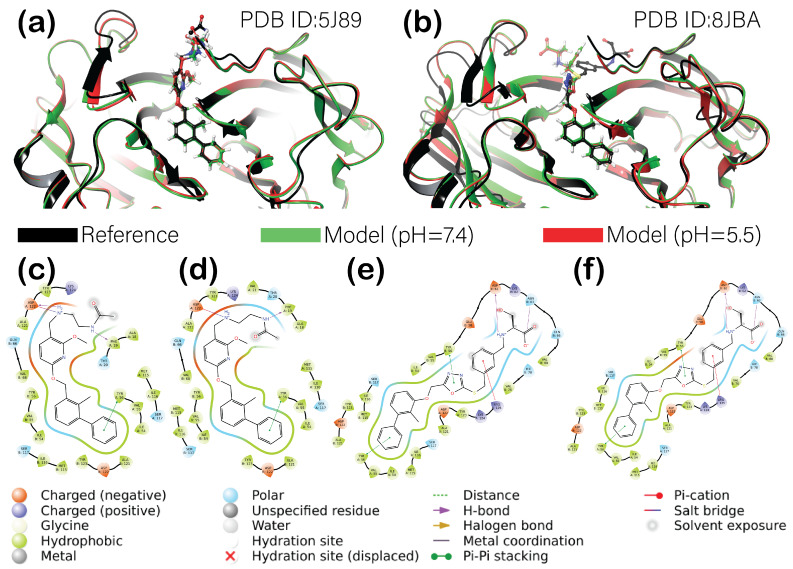
The results of a positive control re-docking and molecular mechanics simulations for PD-L1 complex with small-molecule inhibitors at different pHs: superposition of a reference crystallographic structure with re-docked complexes at two different target pHs for (**a**)—PDB ID:5J89, and (**b**)—PDB ID:8JBA; ligand interaction diagrams for complexes of PD-L1 with re-docked co-crystallized ligands after the molecular mechanics refinement: (**c**)—BMS-202 (PDB ID:5J89) at pH = 7.4, (**d**)—BMS-202 (PDB ID:5J89) at pH = 5.5, (**e**)—LP23 (PDB ID:8JBA) at pH = 7.4, (**f**)—LP23 (PDB ID:8JBA) at pH = 5.5.

One of the best methods to verify the accuracy of a selected molecular modeling approach is the re-docking of a co-crystallized ligand. Two lead compounds were selected for this study: BMS-202 (PDB ID:5J89), a potent inhibitor selective to PD-L1, which promotes its dimerization, and a more recent novel non-arylmethylamine-based inhibitor LP23, which shows higher inhibitory activity than BMS-202 according to [28]. After re-docking and molecular mechanics refinement, both ligands’ diphenyl rings were positioned ideally inside the protein-binding pockets (Figure 3a,b).

**Figure 4:**
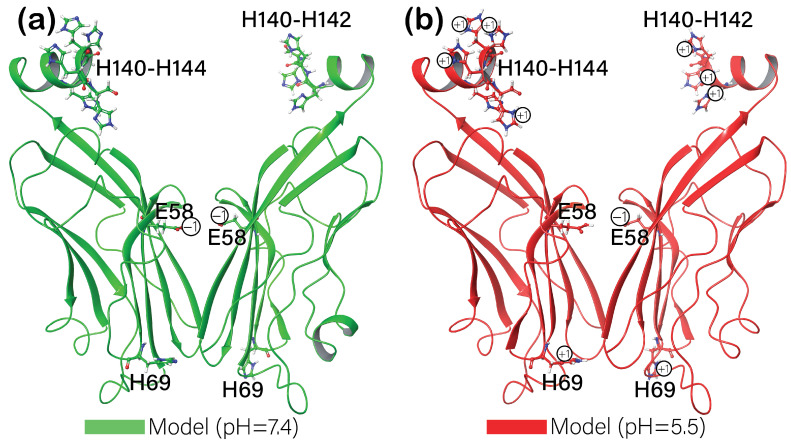
Protein structures and protonation states of the critical residues at two different target pHs: (**a**)—pH = 7.4, (**b**)—pH = 5.5.

The main difference was noticed for the remaining motifs, which were partially exposed to the solvent. The best reproductivity of experimental crystallographic structure was observed for BMS-202, with a slight difference in positions at pH = 5.5 and pH = 7.4. The nature of this difference might be explained by the 2D ligand interaction diagrams (Figure 3c,d) when comparing the interactions between the positively charged amino group of a ligand and the ASP122 residue of a protein. While at a low pH, it only formed a salt bridge between positively charged nitrogen and negatively charged oxygen, at high pH, the additional H-bond was formed. Interestingly, the predicted free binding energy was slightly more negative for the complex at a low pH (−89.77 kcal/mol compared to −87.47 kcal/mol in the case of pH = 7.4). When re-docking the second positive control, ligand LP23, the polar tail connected to the oxadiazole thioether of a ligand showed significant deviation from the reference crystallographic structure. Both high- and low-pH models were re-docked almost identically, forming the same interactions with the protein (Figure 3e,f). Nonetheless, their binding free energies differed slightly with values of −107.88 kcal/mol for a low-pH model and −105.40 kcal/mol for a model with a high pH. A slight difference in free binding energies and binding modes (in the case of BMS-202) for models at different pHs was noticed due to a difference in protein protonation states. At a pH of 7.4, PD-L1 was predicted to have a total charge of −4, while at pH = 5.5, the total charge of a protein was +6. A more detailed analysis of charged residues was needed to elucidate the principal alteration in protonation states. The comparison of protein structures for the two investigated models showed the only difference between the E58, H69, and H140-144 residues’ protonation states (Figure 4). Further on, considering the positive control results, we chose PDB ID:5J89 as a template for our investigations.

### 3.2. Library Screening and ADMET Profiling

A large database of compounds was necessary to increase the chance of finding one with specific properties that satisfied the needs of this project. We performed a virtual screening for 10,305 natural compounds from the Molport database utilizing both models at a standard physiological pH = 7.4 and cancer microenvironment pH = 5.5. The ten best ligands for each model were chosen based on the docking score and binding efficiency. Five of the ten ligands (Table 1, names bolded) scored well for both low- and high-pH conditions. These five were excluded from further investigation as they do not satisfy the requirement of binding specifically to a protein at a low pH. Ligands MolPort-001-741-806, MolPort-005-945-958, MolPort-001-741-210, MolPort-001-740-310, and MolPort-001-742-690 were bound only in conditions similar to the tumor microenvironment; thus, these compounds were selected as potential hits. These compounds were further renamed for simplification as follows: Compound **1** (MolPort-001-741-806), Compound **2** (MolPort-005-945-958), Compound **3** (MolPort-001-741-210), Compound **4** (MolPort-001-740-310), and Compound **5** (MolPort-001-742-690).

There was no significant difference in the docking scores of these compounds. Compound **1** had the lowest docking score of −14.696 kcal/mol, and Compound **5** showed the lowest docking score of −12.914 kcal/mol among the selected ligands. The ligand efficiency may be more reasonable for scoring potent inhibitors. It is known that the higher molecular weight of a drug results in predicting a greater potency due to a larger number of potential interactions. Meanwhile, this is not always the case. The ligand efficiency takes into consideration corrections to the molecular weight of a ligand. Our results showed that Compound **5** had the best ligand efficiency among the top 5 compounds with a value of −0.38 kcal/mol/HA. Within other ligands, Compound **2** showed the highest impact of the Coulomb and H-bond energy (−79.248 kcal/mol and −8.488 kcal/mol).

**Figure 5:**
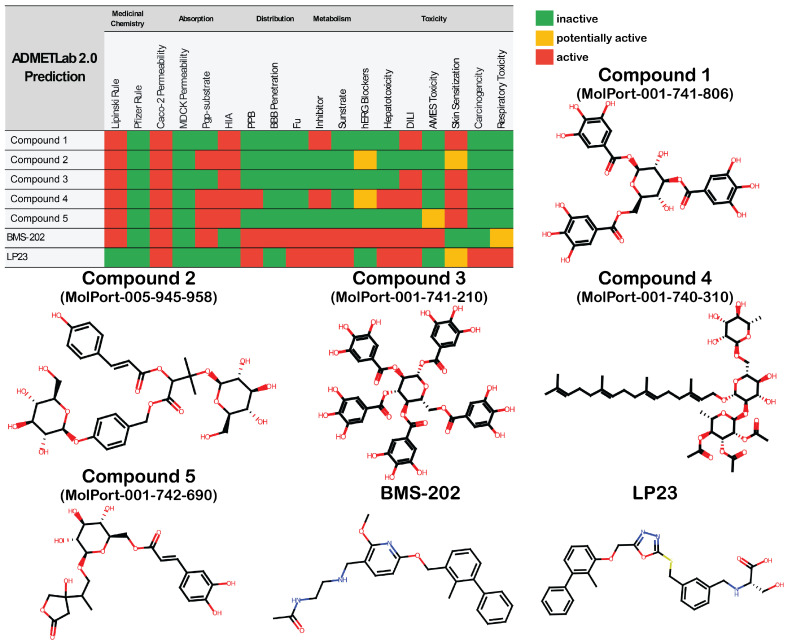
ADMET profiling and 2D structures of top 5 selected compounds and two lead compounds.

ADMET prediction was conducted on the top 5 compounds to evaluate their pharmaceutical properties (Figure 5). Compounds were evaluated to satisfy Lipinski’s Rule of Five, the Pfizer Rule, its adsorption properties, such as Caco-2 permeability, MDCK permeability, P-glycoprotein (Pdp-) substrate, and human intestinal absorption (HIA); distribution, such as plasma protein binding (PPB), blood–brain barrier (BBB) penetration, and fraction unbound (Fu); as well as metabolism and toxicity, such as the human ether-a-go-go related gene (hERG) blockers, hepatotoxicity, drug-induced liver injury (DILI), Ames toxicity (mutagenicity), skin sensitization, carcinogenicity, and respiratory toxicity. These properties were also predicted for the lead compounds BMS-202 and LP23. All compounds except for LP23 failed to satisfy Lipinski’s Rule of Five. However, they all passed the Pfizer Rule. All five potential hits had a low human intestinal absorption, and all studied compounds were predicted not to penetrate human colon adenocarcinoma cell lines (Caco-2), suggesting possible problems with oral admission without further functionalizing a potential drug. BMS-202 was predicted to penetrate the blood–brain barrier, and both BMS-202 and LP23 were shown to have a high plasma protein-binding affinity and a low fraction unbound. The more the drug is bound to proteins in the bloodstream, the less efficiently it can traverse cellular membranes or diffuse. The top five compounds were predicted to have better overall distribution properties, with only Compound **4** having a high plasma protein binding. Both lead compounds were predicted as potential inhibitors and substrates for metabolic processes. Similarly, Compound **1** and Compound **4** were potential metabolism inhibitors. Regarding the predicted toxicities, hit compounds looked more promising than the leads, considering the predicted mutagenicity of BMS-202 and carcinogenicity of LP23. The least toxic among the top five were Compound **2** and Compound **5**.

### 3.3. MM/GBSA

It is well-known that the docking scores have a low agreement with experimental data and may not be efficiently used for scoring accurately. Molecular mechanics with generalized Born and surface area solvation (MM/GBSA) is a popular method used to accurately predict the free energy of binding. The top five hit compounds’ binding poses obtained by docking simulations were refined using the MM/GBSA method in pH 7.4 and 5.5 models. The magnitude of the negative ΔG determines the protein–ligand association extent in molecular mechanics; it can be considered that ΔG determines the stability of any given protein–ligand complex. While the magnitude of ΔG calculated using MM/GBSA cannot be trusted, its comparison usually correlates well with in vitro binding affinities, as shown in [49,50]. These works, among many others, demonstrate significantly elevated free binding energies compared to the experimental results. However, as suggested by Mulakala et al. in [49], the VSGB-2.0 MM/GBSA may be approaching the accuracy required for the absolute binding free energy determination with R^2^ = 0.89 between experimental and predicted affinities for a data set built of several protein targets and a wide range of ligands.

**Figure 6:**
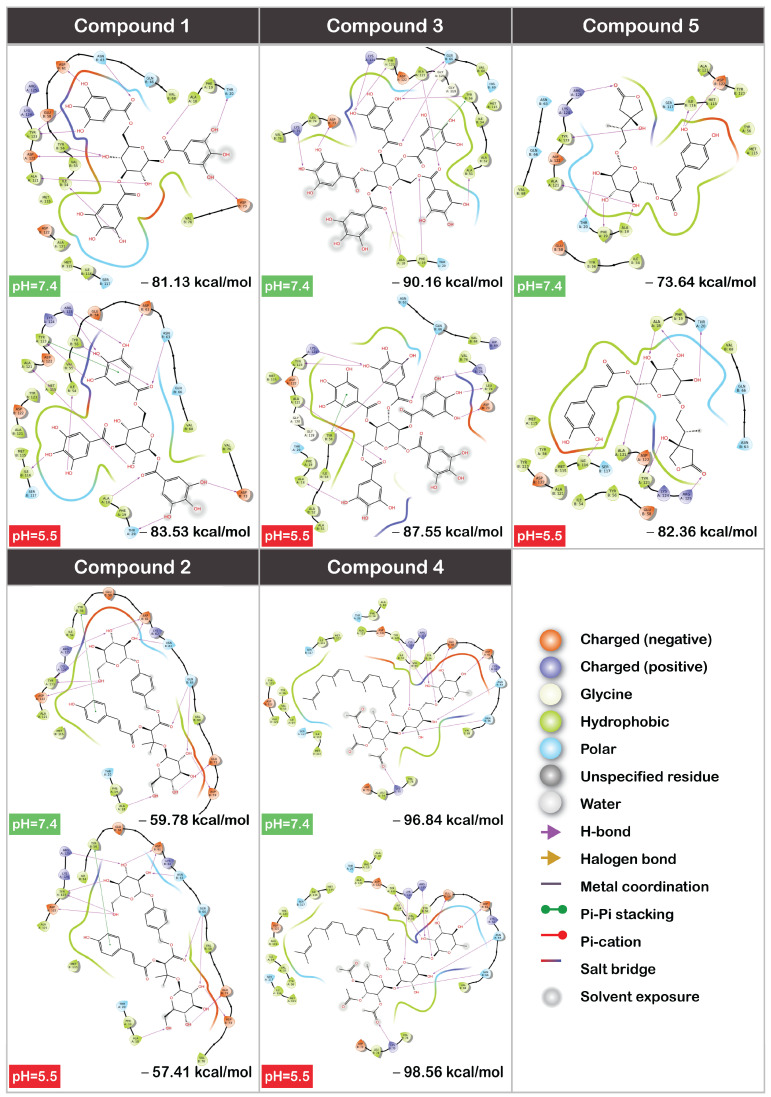
The 2D diagrams of interactions and predicted binding affinities for top 5 selected compounds.

The values predicted that the five ligands bound to PD-L1 did not show significant differences in their binding affinities at different pH values (Figure 6). Forming similar interactions with the protein, Compound **1** complexed with PD-L1 at a pH of 7.4 had a binding free energy of −81.13 kcal/mol, and the value of −83.53 kcal/mol was calculated for a pH of 5.5. The ΔG values of −59.78 kcal/mol and −57.41 kcal/mol at pH = 7.4 and pH = 5.5, respectively, were calculated for Compound **2**, and binding affinities for the Compound **3** complexes were −90.06 kcal/mol and −87.55 kcal/mol. Compound **4** was predicted to have the most negative binding free energy bound to PD-L1. However, no significant difference was noticed between the cases of a pH of 5.5 and physiological conditions for this ligand (−96.84 kcal/mol at a pH of 7.4 and 98.56 kcal/mol at a pH of 5.5). The 2D ligand interaction diagrams also showed no significant difference between binding to a protein at different pH values. The maximal magnitude of difference in binding energies under different pH conditions was 2.51 kcal/mol for Compound **3**. Such a slight difference in the calculated binding energies might be considered a simple noise or error of the method and presumably should not result in a different binding affinity when performing in vitro experiments. It only reflects minor fluctuations in drug-target interactions due to pH variations and could translate to the drug maintaining effective binding across different physiological environments. Nonetheless, this statement must be further verified by an experimental investigation, paying special attention to Compound **4**, which was predicted to have an even higher affinity toward PD-L1 than the potent BMS-202.

In the case of Compound **5**, disregarding the similar ligand interaction diagram, the most remarkable difference in binding affinity was found, with a significantly less negative ΔG in the case of a physiological pH (−73.64 kcal/mol) compared to the case of a low pH 5.5 (−82.36 kcal/mol). It must be noted that at a higher pH, the compound seemed to form more H-bonds. The hydroxyl group from the phenyl ring of a ligand formed two H-bonds (with I116 and D122) at a higher pH, while at a lower pH, it was only bound to I116. Another difference worth mentioning was the H-bond formed by a para-hydroxyl group of a glucose motif. In the case of a higher pH, it donated hydrogen to F19, while at a lower pH, this hydrogen was donated to A18. Despite the complex at pH 7.4 appearing to be more promising in the 2D ligand interaction diagram, quantitatively, it was inferior to the low-pH complex within the H-bond component (−5.54 kcal/mol for pH = 5.5 vs. −5.38 kcal/mol for pH = 7.4), and most importantly, the Coulomb energy component (−73.19 kcal/mol for pH = 5.5 vs. −54.70 kcal/mol for pH = 7.4). The Coulomb energy is the energy associated with the repulsion between charged particles. Thus, the total charge of a protein may play a crucial role in this component’s contribution.

### 3.4. Molecular Dynamics

**Figure 7:**
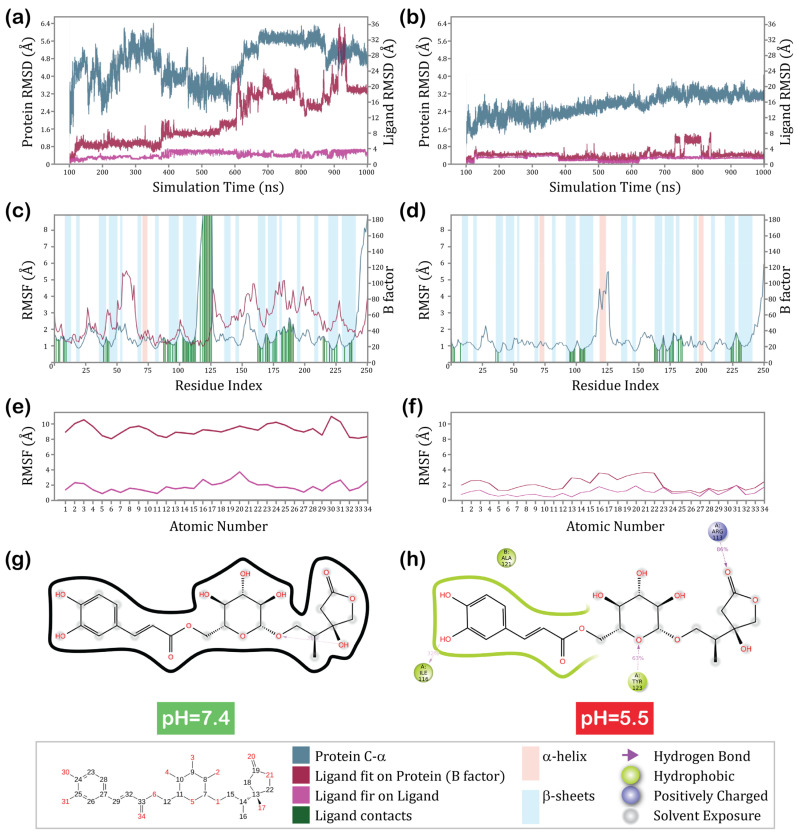
Results of molecular dynamics simulations: protein and ligand RMSD for PD-L1/Compound **5** complexes at (**a**)—pH = 7.4, (**b**)—pH = 5.5; protein RMSF at (**c**)—pH = 7.4, (**d**)—pH = 5.5; ligand RMSF at (**e**)—pH = 7.4, (**f**)—pH = 5.5; and 2D ligand interaction diagram at (**g**)—pH = 7.4, (**h**)—pH = 5.5.

For further evaluation of a hit Compound **5** compound, an extensive 1000 ns simulation was conducted to estimate the stability of the obtained complexes. In conditions of a physiological pH, the complex was shown to be unstable with high protein and ligand root mean square deviations (RMSD) values (Figure 7a). The protein’s RMSD mostly ranged from 2.4 to 6.4, while the ligand’s deviations fit on protein reached as high as 36 Å, with the most significant instability shown after 600 ns. The ligand’s fit on the ligand remained constant throughout the 1000 ns with relatively minimal changes. When tested at the pH of the tumor microenvironment (Figure 7b), the protein RMSD exhibited significantly more stability. The RMSD of the ligand fit on protein also remained extremely stable until a short fragment of deviations between 725 and 825 ns, where an increase by approximately 6 Å was noted. After this brief increase, the ligand returned to its former position for the remainder of the time. The RMSD of the ligand fit on the ligand remained stable throughout the 1000 ns. The root mean square fluctuation (RMSF) calculates individual residue flexibility. It demonstrates how much a particular residue moved during the simulation. The model with a higher pH showed slightly more flexibility and fluctuation across the binding residues up to 3 Å for most of the sequence (Figure 7c). In contrast, for the model with a low pH, these fluctuations did not exceed 2 Å (Figure 7d). Exceptionally high fluctuations were observed between indexes 116–130 and 240–252, corresponding to the sequence residues V130-H144 from the first subunit and V130-H142 from the subunit. These are the helix structures located at the C-terminal of the PD-L1, for which fluctuation is an ordinary event. The significant protein residue fluctuations for a model of pH 7.4 were combined with a very high RMSF of the ligand fit on protein, ranging between 8 and 11 Å across the whole ligand structure (Figure 7e). The ligand fluctuations in lower-pH conditions were significantly lower (Figure 7f), not exceeding 2 Å when fit on the ligand and with a maximum of 4 Å when it was fit on the protein, resulting in a significantly more stable complex. The 2D ligand interaction diagram supported the above observations (Figure 7g,h). Compound **5** left the binding pocket of a protein within 1000 ns simulation at pH 7.4. Conversely, at a pH of 5.5, the results showed the ligand to be very stable in the binding pocket. Several meaningful interactions were demonstrated between the ligand and protein, including a hydrogen bond formed between the double-bonded oxygen of the hydroxyoxolan-2-one fragment and R113 residue (86% of the simulation time), the oxygen of glucose motif and Y123 residue (63% of the simulation time), and between the hydroxyl group of the phenyl ring and I116 amino acid residue (32% of the simulation time). With this apparent difference in affinities, supported by both molecular mechanics and molecular dynamics simulation, Compound **5** could be a potential hit in the search for a drug that would selectively inhibit PD-L1 in cancer microenvironment pH conditions while interacting significantly less with this protein at a normal physiological pH.

## 4. Discussion

The investigation into PD-L1 inhibitors reveals key insights into the effects of pH on binding affinity and stability, offering some understanding of how these factors might be leveraged to enhance cancer immunotherapy. The initial step involved the re-docking of the known PD-L1 inhibitors, BMS-202 and LP23, to validate our computational approach. By achieving a high agreement of a binding pose with known crystal structures, we established a foundation for further accurate analyses. The difference in PD-L1’s charge at different pHs (Figure 4) favored the proposed hypothesis of an attempt to investigate potential inhibitors, which would have a greater affinity toward PD-L1 at a low pH. The comparison of protein structures for the two investigated models revealed that at a physiological pH of 7.4, all histamine amino acid residues were neutral, and glutamic acid was deprotonated. Lowering the pH to 5.5 resulted in the protonation of most histamine residues (H69, H140 (for only one subunit), H141, H142, H143, and H144), which is obvious considering its sidechain pK_A_ value of around 6.04. Interestingly, the glutamic acid residue of the first subunit was also protonated, while E58 of the other subunit remained deprotonated. The sidechain of the glutamic acid has a pK_A_ value of around 4.07, which suggests that the pH used for these models was not low enough to protonate it. However, protein protonation is more complex than simply considering the pK_A_ values of each residue. This complexity arises because of the electrostatic interactions between ionizable groups within the protein and the surrounding solvent, affecting the pK_A_ and these groups’ protonation state.

Our study then progressed to a comprehensive virtual screening of over 10,000 compounds, aiming to discover potential inhibitors that demonstrate selective activity under acidic conditions, typical for cancerous tissues, while showing reduced activity at the physiological pH of healthy cells. Compound **5** emerged as particularly promising of the screened compounds due to its high binding affinity and stability at lower pH levels, as evidenced by molecular dynamics simulations. These simulations were essential in illustrating that Compound **5** fits well within the PD-L1 binding pocket under acidic conditions and maintains its conformation (Supplementary Video S1), suggesting that it could remain effective over longer periods in the tumor microenvironment. Molecular dynamics simulation in normal physiological conditions revealed this complex to be unstable (Supplementary Video S2), which satisfied the desired activity of a potential drug candidate within this hypothesis. Moreover, the assessment of pharmaceutical properties through ADMET profiling provided deeper insights into the pharmacokinetics of Compound **5**, predicting it to be overall less toxic when compared to the known PD-L1 inhibitors, BMS-202 and LP23. Notably, the compound displayed a low potential for hepatotoxicity and no significant carcinogenic risk, which are critical considerations for developing a safe cancer therapy. These properties highlight Compound **5**’s suitability for further preclinical development.

To the best of the authors’ knowledge, limited information is available about this compound in the literature. MolPort-001-742-690, or ([(2R,3S,4S,5R,6R)-3,4,5-trihydroxy-6-[2-(3-hydroxy-5-oxooxolan-3-yl)propoxy]oxan-2-yl]methyl(E)-3-(3,4-dihydroxyphenyl)prop-2-enoate) is a derivative of a hydroxycinnamic acid, found as a metabolite in species such as *Mus musculus* and *Nippostrongylus brasiliensis* according to the ChEBI database. It also might be found in *Rindera graeca* root extracts derived from various culture conditions [51]. Some bioassay data available on the PubChem website indicate that most assays performed (quantitative high-throughput screenings (qHTS) or regular high-throughput screenings (HTS)) Compound **5** was found to be inactive. The only bioassay showing potential activity was a primary fluorescence-based thiol-reactive (MSTI) qHTS assay to identify artifact compounds [52]. This suggests that the compound can bind natural thiols and potentially modify protein cysteine residues. Cytotoxic profiling of annotated libraries using quantitative high-throughput screening revealed this compound to be inactive, suggesting low toxicity and validating the ADMET prediction illustrated in this work. Interestingly, the primary qHTS used to identify gynecologic anti-cancer compounds using libraries of 7914 approved drugs and bioactive compounds showed Compound **5** to be inactive or inconclusive [53]. However, considering that this study aimed to analyze the chemotherapeutic activities of compounds against gynecologic cancer cell lines, it may not necessarily correlate with immunotherapy results due to differences in mechanisms of action. Following this literature search, it is clear that a more detailed experimental investigation of this compound is necessary.

Our findings advocate for the experimental validation and continued exploration of Compound **5** as a hit candidate for targeted cancer immunotherapy. The distinct pH-dependent binding characteristics of this compound could potentially lead to the development of treatments that are effective in the acidic tumor microenvironment and safe for the patient overall.

## 5. Conclusions

Immunotherapeutic treatments for cancer offer significant advantages over traditional treatment options due to their targeted approach and potential for reduced side effects. Our research aimed to discover a safe yet effective natural compound capable of inhibiting the binding of PD-1 to PD-L1, specifically in the acidic conditions typical of the tumor microenvironment (pH of 5.5), without affecting PD-L1 at the normal physiological pH. The goal was to find an inhibitor that would allow T-cells to continue proliferating and fighting cancer cells without inadvertently suppressing the immune response in healthy tissue.

Through extensive in silico methods, including virtual screening, ADMET prediction, MM/GBSA, and molecular dynamics simulations, we analyzed over 10,000 compounds. Our efforts led us to Compound **5** (MolPort-001-742-690), a compound that demonstrated significant potential for further study through in vitro methods to assess its viability as a cancer treatment. Compound 5 showed promising binding affinity and ligand efficiency in virtual screening, while ADMET predictions suggested minimal side effects. Moreover, molecular dynamics simulations revealed significant correlations in binding affinity related to pH levels, indicating that this compound could strongly bind to PD-L1 under acidic, tumor-like conditions and potentially remain bound long enough to exert a therapeutic effect. These data support the further exploration of MolPort-001-742-690 as a targeted, pH-selective cancer therapy, which still needs experimental confirmation.

## Figures and Tables

**Table 1:** Results of the virtual screening against PD-L1 protein at physiological pH and pH = 5.5.

Ligand	Docking Score	Glide Ligand Efficiency	XP GScore	Glide GScore	Glide Evdw	Glide Ecoul	Glide Energy	Glide Einternal	XP HBond
	pH = 7.4								
MolPort-039-339-177	−14.413	−0.257	−14.413	−14.413	−46.675	−32.076	−78.751	19.539	−8.955
MolPort-001-740-898 ^1^	−14.256	−0.324	−14.256	−14.256	−59.246	−16.306	−75.552	18.202	−5.5
MolPort-001-741-409 ^1^	−14.029	−0.319	−14.029	−14.029	−51.465	−24.336	−75.802	11.204	−6.634
MolPort-027-853-642 ^1^	−13.175	−0.388	−13.175	−13.175	−58.105	−16.254	−74.359	0	−4.637
MolPort-042-675-462 ^1^	−13.101	−0.397	−13.101	−13.101	−57.753	−17.173	−74.926	0	−4.487
MolPort-006-668-633	−12.796	−0.284	−12.796	−12.796	−65.269	−18.75	−84.018	17.747	−3.987
MolPort-019-936-738 ^1^	−12.7	−0.302	−12.7	−12.7	−37.706	−21.373	−59.079	0	−5.4
MolPort-019-937-075	−12.664	−0.422	−12.664	−12.664	−48.802	−19.247	−68.049	10.551	−5.187
MolPort-001-741-410	−12.601	−0.286	−12.601	−12.601	−55.576	−23.382	−78.959	14.896	−5
MolPort-044-637-514	−12.574	−0.322	−12.574	−12.574	−46.353	−23.009	−69.362	17.401	−5.133
	pH = 5.5								
MolPort-001-741-806	−14.696	−0.327	−14.696	−14.696	−51.996	−25.228	−77.224	13.831	−7.669
MolPort-001-740-898 ^1^	−14.433	−0.328	−14.433	−14.433	−54.059	−18.633	−72.692	13.624	−5.871
MolPort-042-675-462 ^1^	−13.97	−0.423	−13.97	−13.97	−41.551	−19.12	−60.672	0	−5.731
MolPort-027-853-642 ^1^	−13.85	−0.407	−13.85	−13.85	−57.316	−19.191	−76.507	16.901	−4.977
MolPort-001-741-409 ^1^	−13.809	−0.314	−13.809	−13.809	−56.672	−22.95	−79.622	12.204	−6.214
MolPort-005-945-958	−13.165	−0.263	−13.165	−13.165	−38.363	−40.886	−79.248	16.063	−8.488
MolPort-001-741-210	−13.144	−0.196	−13.144	−13.144	−58.858	−15.814	−74.672	16.758	−7.016
MolPort-001-740-310	−12.987	−0.213	−12.987	−12.987	−77.865	−14.571	−92.436	33.752	−3.641
MolPort-001-742-690	−12.914	−0.38	−12.914	−12.914	−51.647	−17.114	−68.762	19.835	−5.107
MolPort-019-936-738 ^1^	−12.759	−0.304	−12.759	−12.759	−37.839	−21.105	−58.944	0	−5.479

^1^ Five of the ten ligands scored well for both low- and high-pH conditions.

## Data Availability

The dataset is available upon request from the authors.

## Supplementary Materials

The following supporting information can be downloaded at: https://drive.google.com/drive/folders/1PsXOAjVCg_WqTqI9YSz_XGtb9pDjtYaw?usp=drive_link (accessed on 19 June 2024). Video S1: molecular dynamics trajectory of MolPort-001-742-690/PD-L1 complex at pH = 5.5; Video S2: molecular dynamics trajectory of MolPort-001-742-690/PD-L1 complex at pH = 7.4.

## References

[1] Kennedy S.R., Loeb L.A., Herr A.J. (2012). Somatic mutations in aging, cancer, and neurodegeneration. Mech. Ageing Dev..

[2] Kleeff J., Ronellenfitsch U. (2021). Surgical Oncology: Multidisciplinarity to Improve Cancer Treatment and Outcomes. Curr. Oncol..

[3] Anand U., Dey A., Chandel A.K.S., Sanyal R., Mishra A., Pandey D.K., De Falco V., Upadhyay A., Kandimalla R., Chaudhary A. (2023). Cancer chemotherapy and beyond: Current status, drug candidates, associated risks and progress in targeted therapeutics. Genes. Dis..

[4] Chaput G., Sumar N. (2022). Endocrine therapies for breast and prostate cancers. Can. Fam. Physician.

[5] Baskar R., Lee K.A., Yeo R., Yeoh K.-W. (2012). Cancer and Radiation Therapy: Current Advances and Future Directions. Int. J. Med. Sci..

[6] Zhang Y., Zhang Z. (2020). The history and advances in cancer immunotherapy: Understanding the characteristics of tumor-infiltrating immune cells and their therapeutic implications. Cell Mol. Immunol..

[7] Nurgali K., Jagoe R.T., Abalo R. (2018). Editorial: Adverse Effects of Cancer Chemotherapy: Anything New to Improve Tolerance and Reduce Sequelae?. Front. Pharmacol..

[8] Starnes C.O. (1992). Coley’s toxins in perspective. Nature.

[9] Kruger S., Ilmer M., Kobold S., Cadilha B.L., Endres S., Ormanns S., Schuebbe G., Renz B.W., D’haese J.G., Schloesser H. (2019). Advances in cancer immunotherapy 2019—Latest trends. J. Exp. Clin. Cancer Res..

[10] Yang L., Ning Q., Tang S. (2022). Recent Advances and Next Breakthrough in Immunotherapy for Cancer Treatment. J. Immunol. Res..

[11] Mondal M., Guo J., He P., Zhou D. (2020). Recent advances of oncolytic virus in cancer therapy. Hum. Vaccines Immunother..

[12] Apolonio J.S., Gonçalves V.L.d.S., Santos M.L.C., Luz M.S., Souza J.V.S., Pinheiro S.L.R., de Souza W.R., Loureiro M.S., de Melo F.F. (2021). Oncolytic virus therapy in cancer: A current review. World J. Virol..

[13] Propper D.J., Balkwill F.R. (2022). Harnessing cytokines and chemokines for cancer therapy. Nat. Rev. Clin. Oncol..

[14] Lin M.J., Svensson-Arvelund J., Lubitz G.S., Marabelle A., Melero I., Brown B.D., Brody J.D. (2022). Cancer vaccines: The next immunotherapy frontier. Nat. Cancer.

[15] Saxena M., van der Burg S.H., Melief C.J.M., Bhardwaj N. (2021). Therapeutic cancer vaccines. Nat. Rev. Cancer.

[16] Rosenberg S.A., Restifo N.P. (2015). Adoptive cell transfer as personalized immunotherapy for human cancer. Science.

[17] Dudley M.E., Rosenberg S.A. (2003). Adoptive-cell-transfer therapy for the treatment of patients with cancer. Nat. Rev. Cancer.

[18] Park J., Kwon M., Shin E.-C. (2016). Immune checkpoint inhibitors for cancer treatment. Arch. Pharm. Res..

[19] Cha J.-H., Chan L.-C., Li C.-W., Hsu J.L., Hung M.-C. (2019). Mechanisms Controlling PD-L1 Expression in Cancer. Mol. Cell.

[20] Makuku R., Khalili N., Razi S., Keshavarz-Fathi M., Rezaei N. (2021). Current and Future Perspectives of PD-1/PDL-1 Blockade in Cancer Immunotherapy. J. Immunol. Res..

[21] Chamoto K., Hatae R., Honjo T. (2020). Current issues and perspectives in PD-1 blockade cancer immunotherapy. Int. J. Clin. Oncol..

[22] Tang S., Ning Q., Yang L., Mo Z., Tang S. (2020). Mechanisms of immune escape in the cancer immune cycle. Int. Immunopharmacol..

[23] Zhang H., Dai Z., Wu W., Wang Z., Zhang N., Zhang L., Zeng W.-J., Liu Z., Cheng Q. (2021). Regulatory mechanisms of immune checkpoints PD-L1 and CTLA-4 in cancer. J. Exp. Clin. Cancer Res..

[24] Wilkinson E. (2015). Nivolumab success in untreated metastatic melanoma. Lancet Oncol..

[25] Bagcchi S. (2014). Pembrolizumab for treatment of refractory melanoma. Lancet Oncol..

[26] Westin J.R., Chu F., Zhang M., E Fayad L., Kwak L.W., Fowler N., Romaguera J., Hagemeister F., Fanale M., Samaniego F. (2014). Safety and activity of PD1 blockade by pidilizumab in combination with rituximab in patients with relapsed follicular lymphoma: A single group, open-label, phase 2 trial. Lancet Oncol..

[27] Zak K.M., Grudnik P., Guzik K., Zieba B.J., Musielak B., Dömling A., Dubin G., Holak T.A. (2016). Structural basis for small molecule targeting of the programmed death ligand 1 (PD-L1). Oncotarget.

[28] Liu J., Cheng Y., Yuan L., Liu T., Ruan Y., Ren Y., Li L., Jiang S., Xiao Y., Chen J. (2023). Discovery and Crystallography Study of Novel Biphenyl Ether and Oxadiazole Thioether (Non-Arylmethylamine)-Based Small-Molecule PD-1/PD-L1 Inhibitors as Immunotherapeutic Agents. J. Med. Chem..

[29] Sun C., Yin M., Cheng Y., Kuang Z., Liu X., Wang G., Wang X., Yuan K., Min W., Dong J. (2023). Novel Small-Molecule PD-L1 Inhibitor Induces PD-L1 Internalization and Optimizes the Immune Microenvironment. J. Med. Chem..

[30] Cai S., Wang K., Qi Z., Ye K., Zhou X., Jiang S., Zhang K., Zhang X., Wang T. (2023). Design, synthesis, and evaluation of PD-1/PD-L1 small-molecule inhibitors bearing a rigid indane scaffold. Eur. J. Med. Chem..

[31] Sasikumar P.G., Ramachandra M. (2022). Small Molecule Agents Targeting PD-1 Checkpoint Pathway for Cancer Immunotherapy: Mechanisms of Action and Other Considerations for Their Advanced Development. Front. Immunol..

[32] Guo Y., Jin Y., Wang B., Liu B. (2021). Molecular Mechanism of Small-Molecule Inhibitors in Blocking the PD-1/PD-L1 Pathway through PD-L1 Dimerization. Int. J. Mol. Sci..

[33] Almahmoud S., Zhong H.A. (2019). Molecular Modeling Studies on the Binding Mode of the PD-1/PD-L1 Complex Inhibitors. Int. J. Mol. Sci..

[34] Wang F., Ye W., Wang S., He Y., Zhong H., Wang Y., Zhu Y., Han J., Bing Z., Ji S. (2021). Discovery of a new inhibitor targeting PD-L1 for cancer immunotherapy. Neoplasia.

[35] Udhwani T., Mukherjee S., Sharma K., Sweta J., Khandekar N., Nayarisseri A., Singh S.K. (2019). Design of PD-L1 inhibitors for lung cancer. Bioinformation.

[36] Choorakottayil Pushkaran A., Kumaran K., Ann Maria T., Biswas R., Mohan C.G. (2023). Identification of a PD1/PD-L1 inhibitor by structure-based pharmacophore modelling, virtual screening, molecular docking and biological evaluation. Mol. Inform..

[37] Kuang Z., Heng Y., Huang S., Shi T., Chen L., Xu L., Mei H. (2020). Partial Least-Squares Discriminant Analysis and Ensemble-Based Flexible Docking of PD-1/PD-L1 Inhibitors: A Pilot Study. ACS Omega.

[38] Luo L., Zhong A., Wang Q., Zheng T. (2021). Structure-Based Pharmacophore Modeling, Virtual Screening, Molecular Docking, ADMET, and Molecular Dynamics (MD) Simulation of Potential Inhibitors of PD-L1 from the Library of Marine Natural Products. Mar. Drugs.

[39] Boussadia Z., Zanetti C., Parolini I. (2020). Role of microenvironmental acidity and tumor exosomes in cancer immunomodulation. Transl. Cancer Res..

[40] Mori D., Tsujikawa T., Sugiyama Y., Kotani S., Fuse S., Ohmura G., Arai A., Kawaguchi T., Hirano S., Mazda O. (2021). Extracellular acidity in tumor tissue upregulates programmed cell death ligand 1 expression on tumor cells via proton-sensing G protein-coupled receptors. Int. J. Cancer.

[41] Korenchan D.E., Flavell R.R. (2019). Spatiotemporal pH Heterogeneity as a Promoter of Cancer Progression and Therapeutic Resistance. Cancers.

[42] Jacobson M.P., Friesner R.A., Xiang Z., Honig B. (2002). On the Role of the Crystal Environment in Determining Protein Side-chain Conformations. J. Mol. Biol..

[43] Shelley J.C., Cholleti A., Frye L.L., Greenwood J.R., Timlin M.R., Uchimaya M. (2007). Epik: A software program for pK a prediction and protonation state generation for drug-like molecules. J. Comput. Aided Mol. Des..

[44] Lu C., Wu C., Ghoreishi D., Chen W., Wang L., Damm W., Ross G.A., Dahlgren M.K., Russell E., Von Bargen C.D. (2021). OPLS4: Improving Force Field Accuracy on Challenging Regimes of Chemical Space. J. Chem. Theory Comput..

[45] Halgren T.A., Murphy R.B., Friesner R.A., Beard H.S., Frye L.L., Pollard W.T., Banks J.L. (2004). Glide: A New Approach for Rapid, Accurate Docking and Scoring. 2. Enrichment Factors in Database Screening. J. Med. Chem..

[46] Genheden S., Ryde U. (2015). The MM/PBSA and MM/GBSA methods to estimate ligand-binding affinities. Expert Opin. Drug Discov..

[47] Xiong G., Wu Z., Yi J., Fu L., Yang Z., Hsieh C., Yin M., Zeng X., Wu C., Lu A. (2021). ADMETlab 2.0: An integrated online platform for accurate and comprehensive predictions of ADMET properties. Nucleic Acids Res..

[48] Bowers K.J., Chow E., Xu H., Dror R.O., Eastwood M.P., Gregersen B.A., Klepeis J.L., Kolossvary I., Moraes M.A., Sacerdoti F.D. Scalable algorithms for molecular dynamics simulations on commodity clusters. Proceedings of the SC’06: 2006 ACM/IEEE Conference on Supercomputing, Tampa, FL, USA, 11–17 November 2006.

[49] Meador W.E., Kapusta K., Owolabi I., Autry S.A., Saloni J., Kolodziejczyk W., Hammer N.I., Flynt A.S., Hill G.A., Delcamp J.H. (2022). Ultra-Bright Near-Infrared Sulfonate-Indolizine Cyanine- and Squaraine-Albumin Chaperones: Record Quantum Yields and Applications. ChemPhotoChem.

[50] Mulakala C., Viswanadhan V.N. (2013). Could MM-GBSA be accurate enough for calculation of absolute protein/ligand binding free energies?. J. Mol. Graph. Model..

[51] Naliwajski M.R., Wileńska B., Misicka A., Pietrosiuk A., Sykłowska-Baranek K. (2022). HPLC-PDA-ESI-HRMS-Based Profiling of Secondary Metabolites of Rindera graeca Anatomical and Hairy Roots Treated with Drought and Cold Stress. Cells.

[52] Alves V.M., Yasgar A., Wellnitz J., Rai G., Rath M., Braga R.C., Capuzzi S.J., Simeonov A., Muratov E.N., Zakharov A.V. (2023). Lies and Liabilities: Computational Assessment of High-Throughput Screening Hits to Identify Artifact Compounds. J. Med. Chem..

[53] Gorshkov K., Sima N., Sun W., Lu B., Huang W., Travers J., Klumpp-Thomas C., Michael S.G., Xu T., Huang R. (2019). Quantitative Chemotherapeutic Profiling of Gynecologic Cancer Cell Lines Using Approved Drugs and Bioactive Compounds. Transl. Oncol..

